# PCDHGB7 inhibits the progression of triple‐negative breast cancer by suppressing XRCC5/MYC‐mediated ribosome biogenesis

**DOI:** 10.1002/ctm2.70437

**Published:** 2025-09-02

**Authors:** Ming Shan, Hongjie Bi, Hua Sun, Yixin Li, Zhiqi Li, Ziyuan Wang, Jianguo Dong, Shengbo Sang, Lili Ren, Yuguang Ye, Tianzhen Wang, Siniša Volarević, Rui Su, Lei Zhang, Minghui Zhang, Yan He, Guoqiang Zhang, Jing Li, Xiaobo Li

**Affiliations:** ^1^ Department of Breast Surgery Harbin Medical University Cancer Hospital Harbin China; ^2^ Department of Pathology School of Basic Medical Science Harbin Medical University Harbin China; ^3^ Department of Systems Biology Beckman Research Institute of City of Hope Monrovia California USA; ^4^ Department of Gynecology Harbin Medical University Cancer Hospital Harbin China; ^5^ Department of Molecular Medicine and Biotechnology University of Rijeka Faculty of Medicine Rijeka Croatia; ^6^ Department of Oncology Chifeng Municipal Hospital Chifeng China; ^7^ Department of Radiotherapy Harbin Medical University Cancer Hospital Harbin China; ^8^ Center for Chronic Disease Prevention and Control Harbin Medical University Harbin China; ^9^ Key Laboratory of Preservation of Human Genetic Resources and Disease Control Harbin Medical University Ministry of Education Harbin China

To the Editor:

Hyperactivation of ribosome biogenesis plays a crucial role in driving cancer initiation and progression,^1^, ^2^, ^3^, ^4^, ^5^ but the underlying molecular mechanisms are unclear. In this study, we demonstrated that PCDHGB7 negatively regulates ribosome biogenesis in triple‐negative breast cancer (TNBC) by suppressing the XRCC5‐enhanced MYC activity.

By investigating the expression and clinical correlation of three clustered PCDH genes (including 15 *PCDHA* genes, 16 *PCDHB* genes and 22 *PCDHG* genes) in breast cancer, we demonstrated that PCDHGB7 was significantly decreased in TNBC tissues than in normal tissues and other subtypes of breast cancer (Figure 1A,C,D; Figure S1A,B,D) due to its high DNA methylation level (Figure S1E–M). We also showed that lower expression of PCDHGB7 was associated with poor survival outcomes in TNBC patients (Figure 1B,E–H; Figure S1C). Functionally, we demonstrated that PCDHGB7 overexpression significantly decreased proliferation and metastasis, whereas PCDHGB7 knockdown enhanced the proliferation and metastasis of TNBC cells both in vivo and in vitro (Figure 1I–P; Figure S2A–K).

**FIGURE 1:**
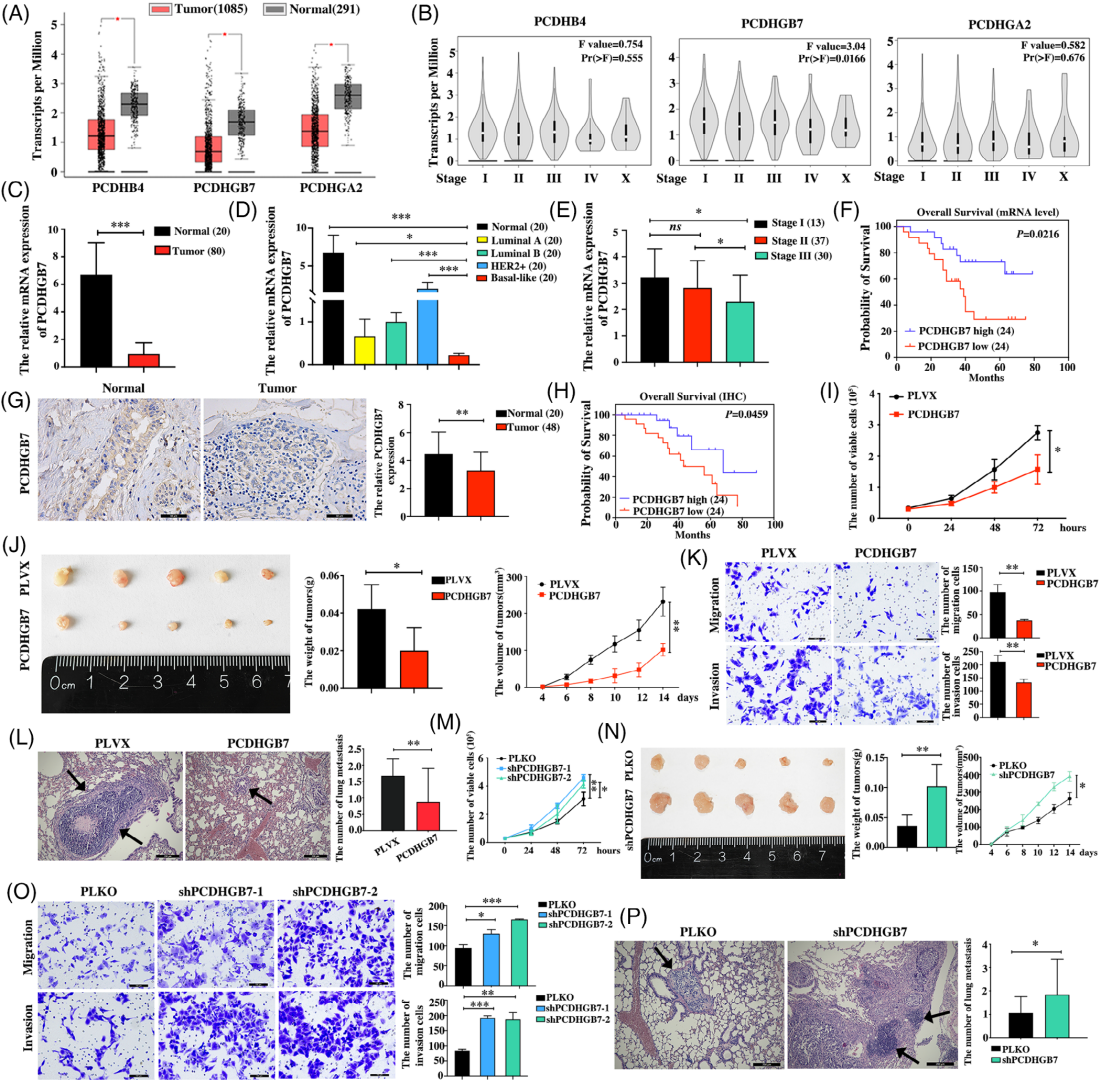
*PCDHGB7* is downregulated and serves as a tumour suppressor in TNBC. (A) The levels of PCDHB4, PCDHGB7 and PCDHGA2 mRNA expression in breast cancer compared to normal breast tissues were investigated using the GEPIA database (tumour tissues *n* = 1085, normal tissues *n* = 291; *p < .05). (B) The levels of PCDHB4, PCDHGB7 and PCDHGA2 mRNA expression in various stages of breast cancer were analysed using the GEPIA database (*p *< .05). (C) PCDHGB7 mRNA expression was examined in 80 breast cancer tissue samples and 20 normal tissue samples using RT‐qPCR (***p < .001). (D) PCDHGB7 mRNA expression in normal breast tissues and various breast cancer subtypes was quantitatively analysed by RT‐qPCR (*n *= 20 for each group; *p < .05, ***p < .001). (E) PCDHGB7 mRNA expression levels were assessed across different stages of breast cancer by RT‐qPCR in a study cohort comprising 80 breast cancer tissue samples (*p < .05, ns = no significant difference). (F) Kaplan–Meier survival analysis was performed to evaluate the prognostic significance of PCDHGB7 expression in TNBC patients (*p *= .0216). (G) PCDHGB7 protein expression in 48 paraffin‐embedded TNBC tissues and 20 adjacent normal tissues was determined by IHC assay (magnification: ×400; scale bars = 50 µm; **p < .01). (H) Kaplan–Meier plots were generated to investigate the relationship between the overall survival of TNBC patients and PCDHGB7 expression with an IHC assay (*p *= .0459). (I) The impact of PCDHGB7 overexpression on the proliferation of Hs578T cells was assessed with the MTT assay (*p < .05). (J) The impact of PCDHGB7 overexpression on the proliferation of Hs578T cells in a xenograft mouse model was assessed by measuring the tumour weight and volume (*p < .05, **p < .01). (K) The impact of PCDHGB7 overexpression on the migratory and invasive capabilities of Hs578T cells was evaluated by Transwell assay (magnification: ×200; scale bars = 100 µm; **p < .01). (L) The impact of PCDHGB7 overexpression on lung metastasis was assessed in a model in which Hs578T cells were injected into mice via the tail vein (magnification: ×100; scale bars = 200 µm; the black arrow indicates the metastatic foci; **p < .01). (M) The impact of PCDHGB7 knockdown on the proliferation of Hs578T cells was assessed by the MTT assay (*p < .05, **p < .01). (N) The impact of PCDHGB7 knockdown on the growth of Hs578T cells in a xenograft mouse model was assessed by measuring the weight and volume of the tumours (*p < .05, **p < .01). (O) The effect of PCDHGB7 knockdown on the migration and invasion of Hs578T cells was examined (magnification: ×200; scale bars = 100 µm; *p < .05, **p < .01, ***p < .001). (P) The effect of PCDHGB7 knockdown on lung metastasis was assessed in a model in which Hs578T cells were injected into mice via the tail vein (magnification: ×100; scale bars = 200 µm; the black arrow indicates the metastatic foci; *p < .05). All data are presented as means ± SD.

To elucidate the mechanisms by which PCDHGB7 inhibits TNBC progression, we employed mass spectrometry (MS) to identify 219 proteins that were upregulated and 231 proteins that were downregulated following *PCDHGB7* knockdown in TNBC cells (Figure S3A; Table S2). Gene Ontology analysis revealed that a subset of proteins upregulated upon *PCDHGB7* knockdown were enriched in pathways related to ribosome biogenesis and rRNA processing (Figure 2A; Table S3), indicating that PCDHGB7 may negatively regulate ribosome biogenesis in TNBC cells. Polysome profiling revealed that PCDHGB7 overexpression decreased, whereas PCDHGB7 knockdown increased the number of ribosomes in TNBC cells (Figure 2B,C; Figure S3B,C). RT‐qPCR results showed that the levels of 47S pre‐rRNA, 28S, 18S and 5.8S rRNA were significantly decreased upon PCDHGB7 overexpression, but increased upon PCDHGB7 knockdown in both TNBC cell lines (Figure S3D,E,G,H). ChIP experiment showed PCDHGB7 overexpression decreased Pol I occupancy in TNBC cells (Figure 2D; Figure S3F). Moreover, the AHA and SUnSET assays consistently demonstrated that PCDHGB7 depletion significantly increased, whereas PCDHGB7 overexpression decreased protein synthesis in both TNBC cells (Figure 2E–G; Figure S3I,J). Consistent with these findings, we demonstrated that the translation efficacy and number of ribosomes in TNBC cells were significantly greater than those in immortal human breast cells (Figure 2H,I). Additionally, the area of AgNOR staining in TNBC tissues was larger and darker than that in normal breast tissues (Figure 2J,K), and a high staining index predicts a poor prognosis in TNBC patients (Figure 2L). Our findings highlight that increased ribosome biogenesis and increased protein synthesis are characteristic features of TNBC, and that PCDHGB7 inhibits these processes.

**FIGURE 2:**
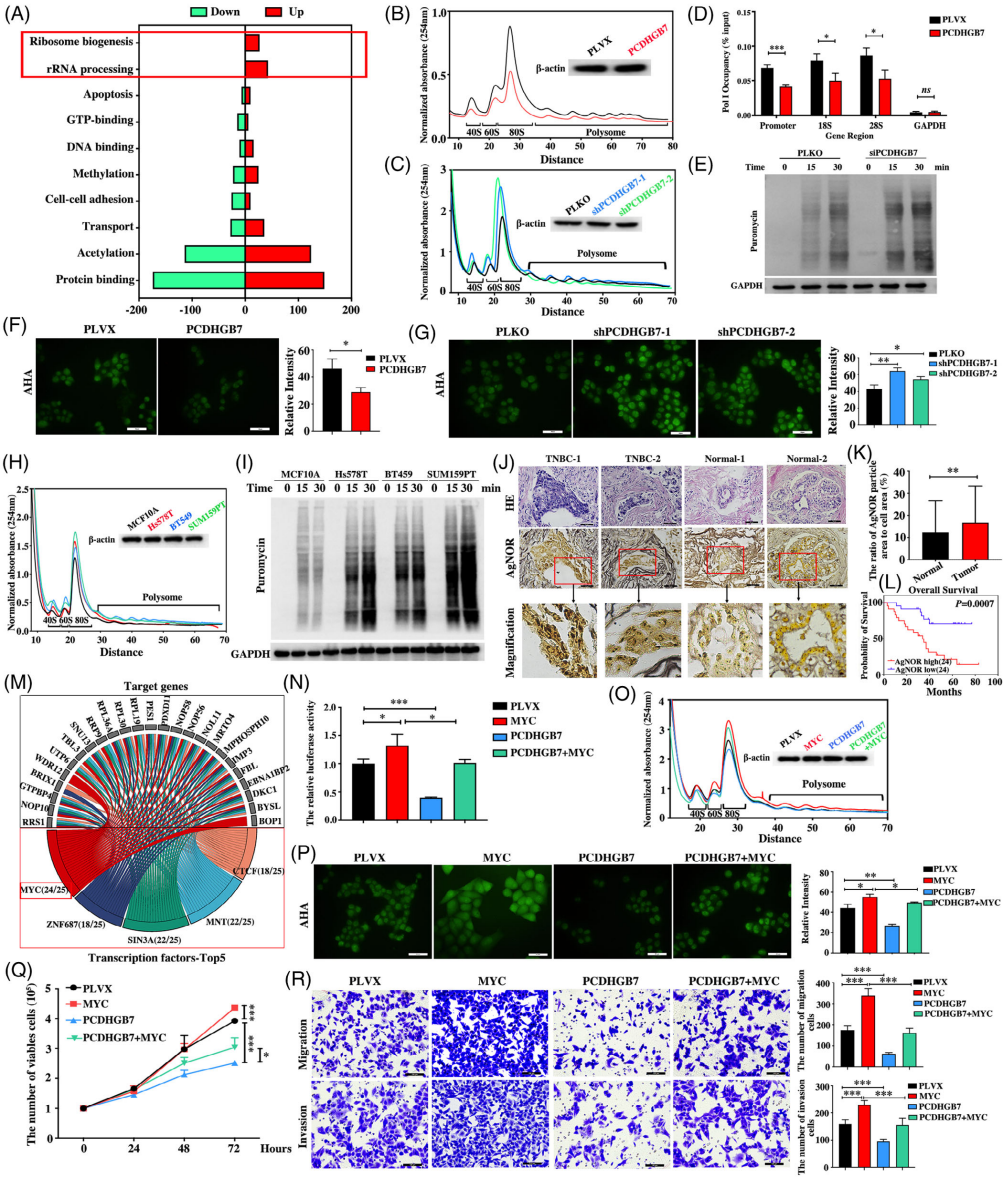
PCDHGB7 suppresses the ribosome biogenesis and MYC activity in TNBC. (A) Functional enrichment analysis of proteins significantly altered upon PCDHGB7 knockdown by using Gene Ontology analysis. (B and C) Polysome profiling assay was performed in PCDHGB7‐overexpression or PCDHGB7‐knockdown Hs578T cells. (D) ChIP assay was used to detect the change of PolI occupancy over different regions of the rDNA in Hs578T‐PCDHGB7 overexpression cells and control cells (*p < .05, ***p < .001, ns = no significant difference). (E) SUnSET assays were used to detect the protein synthesis in PCDHGB7‐overexpression or PCDHGB7‐knockdown Hs578T cells. (F and G) AHA assay was used to detect protein synthesis in PCDHGB7‐overexpression or PCDHGB7‐knockdown Hs578T cells (magnification: ×400; scale bars = 50 µm; *p < .05, **p < .01). (H) Polysome profiling was used to detect the number of ribosomes in MCF10A and TNBC cells. (I) The SUnSET assay was used to detect protein synthesis in MCF10A and TNBC cells. (J) Representative nuclei and nucleoli in TNBC tissues and adjacent normal tissues detected by HE and AgNOR staining, respectively (magnification: ×400; scale bars = 50 µm). (K) Quantitative analysis of the nucleolus area in TNBC tissues and adjacent normal tissues as detected by AgNOR staining (normal *n* = 20, tumour *n* = 48; **p < .01). (L) Kaplan–Meier plots were generated to investigate the relationship between the overall survival of patients and the nucleolar area in TNBC patients (*p *= .0007). (M) The top five transcription factors potentially binding to the promoter region of 25 PCDHGB7 negatively regulated target genes were identified by searching the TFMapper database. (N) Dual‐luciferase reporter assay was used to detect the effect of PCDHGB7 overexpression on MYC transcription activity (*p < .05, ***p < .001). (O) Polysome profiling assay was performed to detect the effect of MYC restoration on PCDHGB7‐mediated suppression of ribosome numbers in Hs578T cells. (P) AHA assay was used to detect the effect of MYC restoration on PCDHGB7‐suppressed protein synthesis in Hs578T cells (magnification: ×400; scale bars = 50 µm; *p < .05, **p < .01). (Q) The effect of MYC restoration on the PCDHGB7‐mediated inhibition of Hs578T cell proliferation, as determined by MTT assay. (*p < .05, ***p < .001). (R) The effects of MYC restoration on the PCDHGB7‐mediated suppression of Hs578T cell migration and invasion, as determined by a Transwell assay (magnification: ×200; scale bars = 100 µm; ***p < .001). All data are presented as means ± SD.

By analysing the TCGA dataset, we further revealed a negative correlation between *PCDHGB7* mRNA level and the mRNA levels of more than half of the genes (25 out of 49) that regulate ribosome biogenesis in breast cancer (Figure S4A; Table S3). We then explored the transcription factors that potentially regulate the transcription of these 25 genes using the TFMapper database, and we revealed that most of these genes (24 out of 25) were potentially regulated by the transcription factor MYC (Figure 2M), which is a master regulator that enhances ribosome biogenesis and translation.^6^, ^7^ Using a dual‐luciferase assay, we showed that PCDHGB7 indeed negatively regulated MYC transcriptional activity (Figure 2N). Further studies indicated that PCDHGB7 overexpression not only reversed MYC‐induced changes in ribosome number, rRNA processing and protein synthesis but also mitigated MYC‐triggered cell proliferation, migration and invasion in TNBC cells (Figure 2O–R; Figure S4B–N).

Through co‐IP followed by MS, we identified 307 proteins that interact with PCDHGB7 (Table S4). Although PCDHGB7 did not directly interact with MYC (Figure S5A), a total of 220 PCDHGB7‐interacting proteins potentially interact with MYC (Figure S5B) according to the Biogrid database (Tables S5 and S6). Gene annotation revealed that some of these proteins that interact with both PCDHGB7 and MYC are associated with ribosome biogenesis (Figure S5C). In particular, x‐ray repair cross‐complementary 5 (XRCC5), which is a well‐recognised oncogene expressed in various cancer types,^8^, ^9^, ^10^ may simultaneously participate in ribosomal biogenesis and rRNA processing (Figure 3A). The direct interaction between XRCC5 and PCDHGB7 or MYC in Hs578T cells was confirmed with PLA (Figure 3B). Moreover, a docking assay revealed that the amino acid sites on XRCC5 that interact with PCDHGB7 are very close to those that interact with MYC (Figure S5D). Additional dual‐luciferase assay revealed that XRCC5 significantly increased the activity of MYC, but blocked the inhibitory effect of PCDHGB7 on the MYC activity (Figure 3C). Therefore, PCDHGB7 suppressed MYC transcriptional activity through competitively interacting with XRCC5 in TNBC.

**FIGURE 3:**
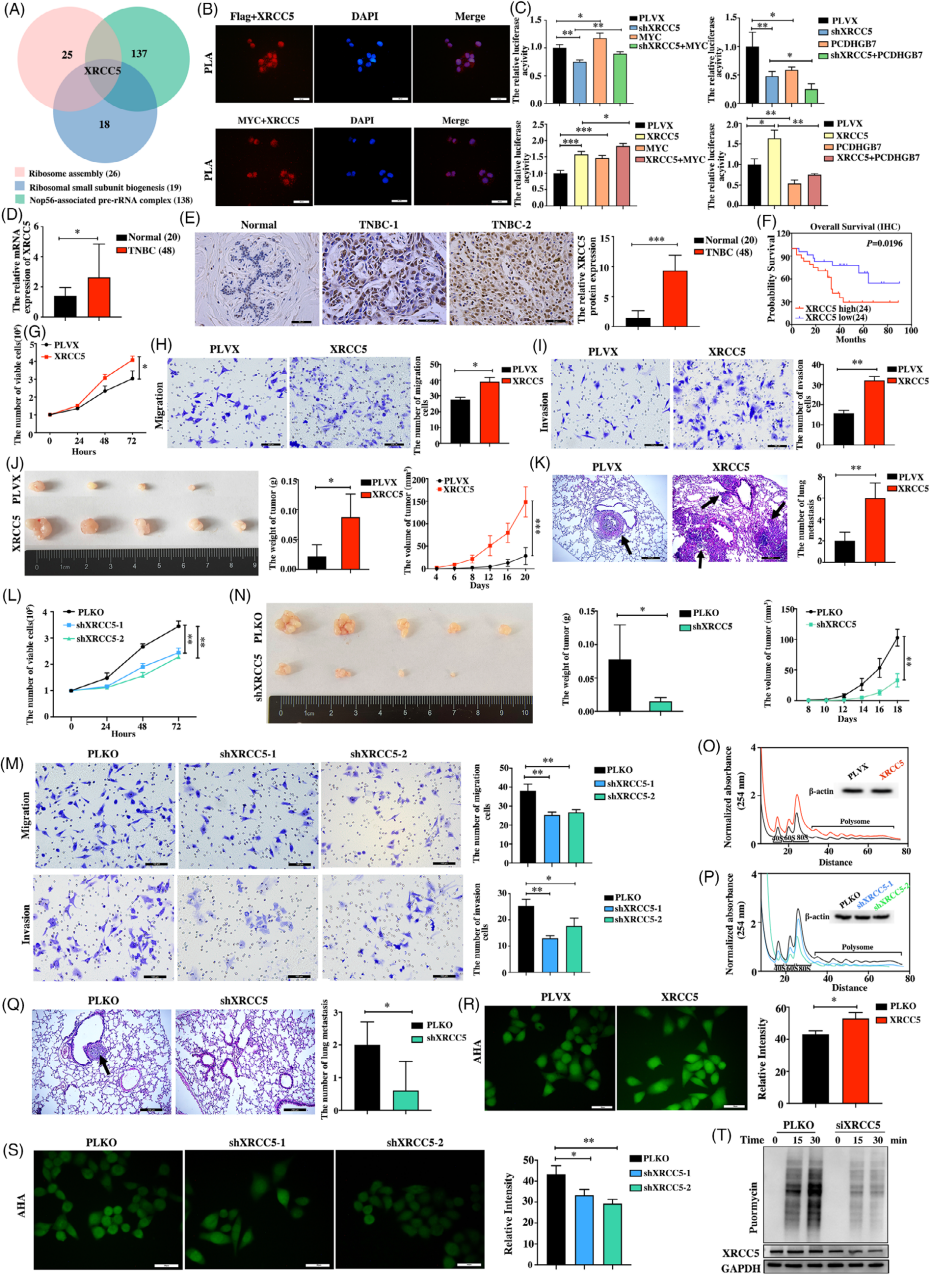
PCDHGB7 suppresses MYC activity by competitively inhibiting XRCC5–MYC interaction in TNBC cells. (A) Cross‐analysis of the proteins that regulate ribosome assembly, ribosomal subunit biogenesis and the Nop56‐associated pre‐rRNA complex. (B) The interactions of PCDHGB7 with XRCC5, MYC and XRCC5 were detected by PLA assay. (C) Dual‐luciferase reporter assay was used to detect the effects of XRCC5 overexpression or knockdown on the MYC transcriptional activity (*p < .05, ** *p *< .01, ***p < .001), and the effects of XRCC5 overexpression or knockdown on PCDHGB7‐suppressed MYC transcriptional activity (*p < .05, **p < .01). (D) XRCC5 expression in 48 paraffin‐embedded TNBC tissues and 20 adjacent normal tissues at the mRNA level was detected by RT‐qPCR (*p < .05). (E) XRCC5 protein expression in 48 paraffin‐embedded TNBC tissues and 20 adjacent normal tissues was determined with the IHC assay (magnification: ×400; scale bars = 50 µm; ***p < .001). (F) Kaplan–Meier plots were used to investigate the relationship between the overall survival of TNBC patients and XRCC5 expression by the IHC assay (*p *= .0196). (G) The effect of XRCC5 overexpression on the proliferation of Hs578T cells was measured by MTT assay (*p < .05). (H and I) The effect of XRCC5 overexpression on the migration and invasion of Hs578T cells was examined with a Transwell assay (magnification: ×200; scale bars = 100 µm; *p < .05, **p < .01). (J) The effect of XRCC5 overexpression on the growth of Hs578T cells in a xenograft mouse model was evaluated by measuring the weight and volume of tumours (*p < .05, ***p < .001). (K) The effect of XRCC5 overexpression on the lung metastasis of Hs578T cells was detected with a tail vein injection model (magnification: ×100; scale bars = 200 µm; the black arrow indicates the metastatic foci; **p < .01). (L) The effect of XRCC5 knockdown on the proliferation of Hs578T cells was measured by MTT assay (**p < .01). (M) The effect of XRCC5 knockdown on the migration and invasion of Hs578T cells was examined with a Transwell assay (magnification: ×200; scale bars = 100 µm; *p < .05, **p < .01). (N) The effects of XRCC5 knockdown on the growth of Hs578T cells in a xenograft mouse model were evaluated based on tumour weight and volume (*p < .05, **p < .01). (O and P) Polysome profiling assay was conducted on Hs578T cells following either overexpression or knockdown of XRCC5. (Q) The effect of XRCC5 knockdown on the lung metastasis of Hs578T cells was detected in a tail vein injection model (magnification: ×100; scale bars = 200 µm; the black arrow indicates the metastatic foci; *p < .05). (R and S) AHA assay was used to investigate protein synthesis in Hs578T cells upon XRCC5 overexpression or knockdown (magnification: ×400; scale bars = 50 µm; *p < .05, ** *p *< .01). (T) SUnSET assays were used to detect the protein synthesis in Hs578T cells upon XRCC5 knockdown. All data are presented as means ± SD.

Using our in‐house paraffin‐embedded tissue cohort, we not only confirmed the high expression of XRCC5 in TNBC tissues compared with normal breast tissues (Figure 3D‐E) but also demonstrated that elevated XRCC5 levels predict poor survival outcomes in TNBC patients (Figure 3F). We further showed that XRCC5 overexpression significantly increased, whereas XRCC5 knockdown decreased TNBC cell proliferation and metastasis in vitro (Figure 3G–I,L,M; Figure S5E–N) and in vivo (Figure 3J,K,N,Q). Furthermore, we showed that XRCC5 overexpression notably increased, whereas XRCC5 knockdown decreased the number of ribosomes in TNBC cells (Figure 3O,P; Figure S6A,B), and XRCC5 overexpression significantly increased the levels of 47S pre‐rRNA, 28S, 18S and 5.8S rRNA in both TNBC cell lines, whereas XRCC5 knockdown decreased the levels of these rRNA (Figure S6C–F). Additionally, we showed that overexpression of XRCC5 promoted, whereas XRCC5 knockdown suppressed protein synthesis in TNBC cells, as detected by the AHA and SUnSET assays (Figure 3R–T; Figure S6G,H). These results demonstrate that XRCC5 promotes TNBC progression and facilitates ribosome biogenesis in TNBC cells.

A rescue assay demonstrated that knockdown of MYC not only attenuated the promotional effect of XRCC5 on promoting the proliferation, migration and invasion of both TNBC cell lines (Figure 4A–C; Figure S7A–G) but also interfered with effect of XRCC5 on increasing the number of ribosomes, rRNA processing and protein synthesis in TNBC cells (Figure 4D–F; Figure S7H–J). In addition, we found that XRCC5 knockdown not only decreased the proliferation, migration and invasion induced by PCDHGB7 knockdown (Figure 4G–I; Figure S7K–Q) but also reduced the number of ribosomes, rRNA processing and protein synthesis mediated by PCDHGB7 knockdown in TNBC cells (Figure 4J–L; Figure S7R–T). Conclusively, we demonstrated that PCDHGB7 negatively regulates ribosome biogenesis and TNBC progression by suppressing XRCC5‐enhanced MYC activity.

**FIGURE 4:**
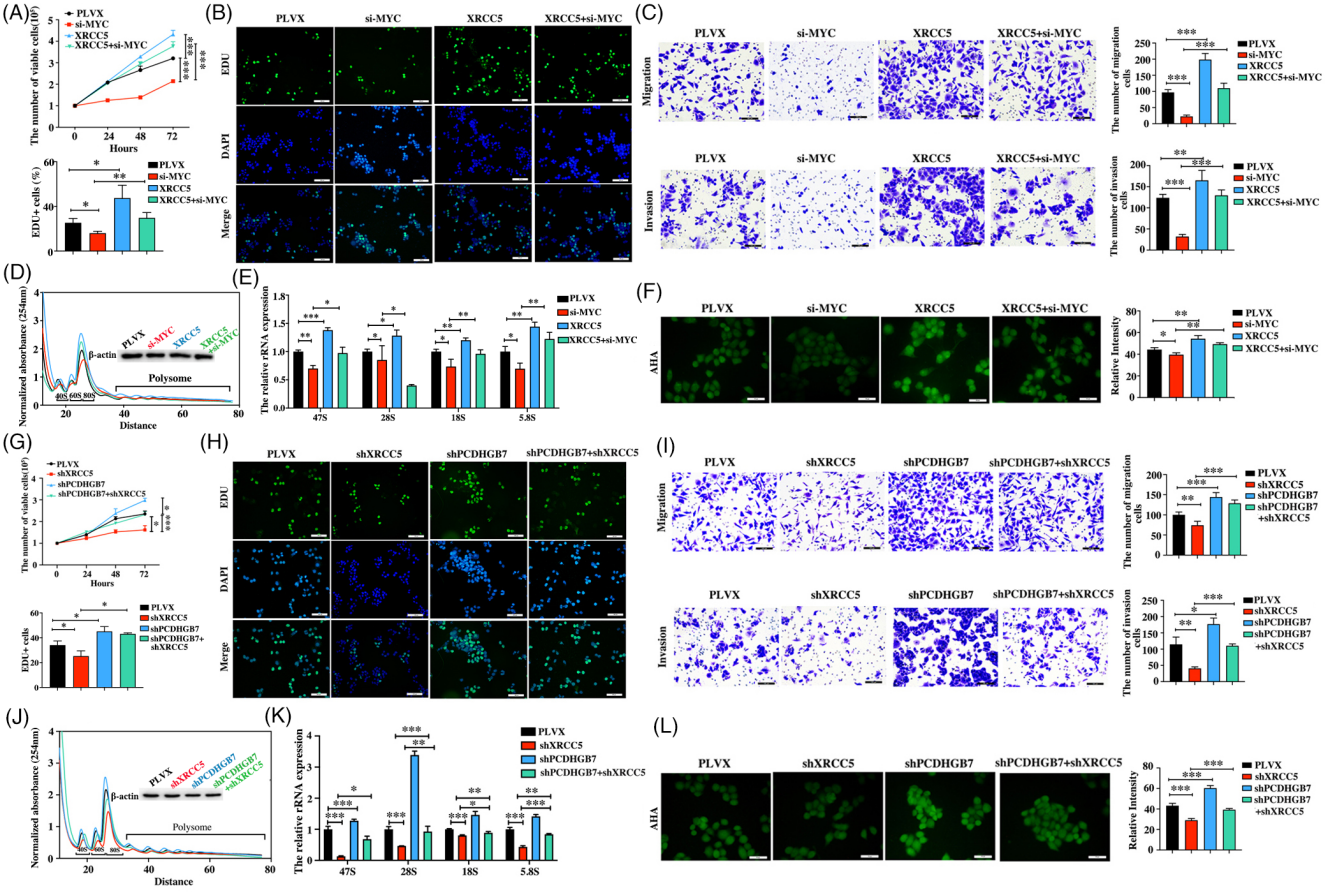
PCDHGB7 inhibits TNBC progression and ribosome biogenesis by suppressing XRCC5‐mediated induction of MYC activity. (A) The effect of MYC knockdown on the XRCC5‐induced proliferation of Hs578T cells, as detected by MTT assay (***p < .001). (B) The impact of MYC knockdown on XRCC5‐induced proliferation in Hs578T cells was evaluated by EDU assay (magnification: ×200; scale bars = 100 µm; *p < .05, **p < .01). (C) The effect of MYC knockdown on the XRCC5‐induced migration and invasion in Hs578T cells was assessed by Transwell assay (magnification: ×200; scale bars = 100 µm; **p < .01, ***p < .001). (D) Polysome profiling assay was performed to investigate the impact of MYC knockdown on the XRCC5‐mediated induction of ribosome biogenesis in Hs578T cells. (E) RT‐qPCR was used to determine the effect of MYC knockdown on the XRCC5‐mediated induction of 47S pre‐rRNA and 28S, 18S and 5.8S rRNA in Hs578T cells (*p < .05, **p < .01, ***p < .001). (F) AHA assay was used to determine the effect of MYC knockdown on the XRCC5‐mediated induction of nascent peptide synthesis in Hs578T cells (magnification: ×400; scale bars = 50 µm; *p < .05, **p < .01). (G) The effect of XRCC5 knockdown on PCDHGB7 knockdown‐induced proliferation of Hs578T cells, as detected by MTT assay (*p < .05, ***p < .001). (H) The impact of XRCC5 knockdown on PCDHGB7 knockdown‐induced proliferation of Hs578T cells as detected by EDU assay (magnification: ×200; scale bars = 100 µm; *p < .05). (I) The effect of XRCC5 knockdown on PCDHGB7 knockdown‐induced migration and invasion of Hs578T cells, as detected by Transwell assay (magnification: ×200; scale bars = 100 µm; *p < .05, **p < .01, ***p < .001). (J) Polysome profiling assay was performed to determine the effect of XRCC5 knockdown on the PCDHGB7 knockdown‐induced ribosome biogenesis in Hs578T cells. (K) RT‐qPCR was used to detect the effect of XRCC5 knockdown on the PCDHGB7 knockdown‐induced production of 47S pre‐rRNA and 28S, 18S and 5.8S rRNA in Hs578T cells (*p < .05, **p < .01, ***p < .001). (L) AHA assay was used to determine the effect of XRCC5 knockdown on the PCDHGB7 knockdown‐induced protein synthesis in Hs578T cells (magnification: ×400; scale bars = 50 µm; ***p < .001). All data are presented as means ± SD.

## 1 MATERIALS AND METHODS

### 1.1 Tissue samples

We procured 80 paraffin‐embedded tumour tissues, subdivided into 20 basal‐like subtype, 20 luminal A subtype, 20 luminal B subtype and 20 HER2^+^ subtype. Additionally, we obtained 20 adjacent normal tissues and 48 TNBC clinical samples with survival data. These samples were collected from patients diagnosed with breast cancer between January 2015 and January 2023 at Harbin Medical University Cancer Hospital. All patients underwent surgical resection, with the diagnostic criteria adhering to the World Health Organization Classification Scheme. The utilisation of these tissues received approval from the Institutional Ethics Committee of Harbin Medical University.

### 1.2 DNA isolation and bisulfite treatment

Genomic DNA was procured from paraffin sections and blood specimens utilising the TIANamp DNA kit, following the manufacturer's guidelines (Catalogue number DP340 for FFPE and DP304‐3 for blood, Tiangen Biochemical Technology Co., Ltd.). The extracted DNA was both quantitatively and qualitatively assessed via a Nanodrop ND‐1000 spectrophotometer. Sodium bisulfite treatment of the genomic DNA led to the transformation of unmethylated cytosine residues into uracil, while the methylated cytosines at CpG sites remained unaltered. Post modification of the genomic DNA using the EZ‐DNA‐Methylation‐GOLD kit (Catalogue number D5006, Zymo Research), in accordance with the manufacturer's protocol, the bisulfite‐treated DNA was preserved at −20°C until further processing.

### 1.3 Cell culture

The four triple‐negative breast cancer cell lines Hs578T (RRID: CVCL_0332), BT549 (RRID: CVCL_1092), MDA‐MB‐468 (RRID: CVCL_0419), SUM159PT (RRID: CVCL_5423) and normal human breast epithelial cell line MCF10A (RRID: CVCL_0598) were procured from the Cell Library of the Chinese Academy of Sciences (Shanghai, China). Both Hs578T and MDA‐MB‐468 cells, along with 293T cells (RRID: CVCL_0063), were diligently maintained in Dulbecco's Modified Eagle's Medium (DMEM, Sigma‐Aldrich). In contrast, SUM159PT and BT549 cells were preserved in RPMI‐1640 medium. All media were enriched with 10% fetal bovine serum and 1% penicillin‐streptomycin. MCF10A cells were maintained in MCF10A Cell Complete Medium (Procell). The cells were consistently incubated at 37°C in a humidified atmosphere with a 5% CO_2_ concentration. All cell lines were authenticated with STR profiling and tested negative for *Mycoplasma*.

### 1.4 Generation of stable cell line construction

The full‐length coding sequences (CDSs) of PCDHGB7, along with a flag tag, were subcloned into the PLVX plasmid. Two shRNAs targeting PCDHGB7 were subcloned into the PLKO plasmid, with target sequences of 5′‐CTAAGGCGGTCAGTACCAAG‐3′ (sh1) and 5′‐CTACAAGCTAGTAACAGATG‐3′ (sh2). Cell transfection with shRNA and the overexpression vector was performed as per the manufacturer's guidelines. In brief, lentivirus production was accomplished in six‐well plates by transfecting 293T cells with 3 µg of the transfer vector, 3 µg of PsPax2 and 1.3 µg of pMD2.G plasmids using 10 µL of lipofectamine. After 48 h, the viral supernatant was introduced into Hs578T and BT549 cells, which were subsequently harvested for further assays. For the generation of stable XRCC5 overexpression and knockdown cells, full‐length XRCC5 coding sequences and two XRCC5 shRNAs (sh1 sequence: 5′‐TGAAGATGGACCTACAGCTAA‐3′ and sh2 sequence: 5′‐CGCTTTAA CAACTTCCTGAAA‐3′) were transfected into Hs578T and BT549 cells, following the same protocol as above.

### 1.5 Cell viability

Cell proliferation was evaluated using the MTT assay (3‐(4, 5‐dimethylthiazol‐2‐yl)‐2,5‐diphenyltetrazolium bromide, MTT). Cells were plated at a density of 5000 cells per well in 96‐well plates. After 24, 48, and 72 h post‐seeding, the cells were incubated with MTT reagent for 4 h, followed by optical density (OD) measurement using a microplate reader. All experiments were conducted in triplicate.

### 1.6 5‐Ethynyl‐2′‐deoxyuridine

Cell proliferation was assessed using 5‐ethynyl‐2′‐deoxyuridine (EDU) staining as per the protocol outlined below. In brief, Hs578T and BT549 cell lines were plated at a density of 5 × 10^4^ cells per well in 24‐well plates. Subsequently, these cells were treated with the EDU assay kit (Beyotime Biotechnology) for a duration of 2 h, followed by fixation with 4% paraformaldehyde for 30 min at ambient temperature. The nuclei were then stained with Hoechst dye. EDU‐positive cells were visualised and quantified using a fluorescence microscope (Nikon Company).

### 1.7 L‐AHA assay

The Click‐iT L‐azidohomoalanine (AHA) assay was performed in accordance with the guidelines provided in the AHA kit (Thermo Fisher Scientific). The procedure was succinctly outlined as follows: cells, cultured in a methionine‐free medium, were incubated with the AHA amino acid analogue for 30 min. Subsequently, the cells were fixed, permeabilised and labelled with Alexa Fluor 488 azide. The cell nuclei were stained with DAPI and visualised under a fluorescence microscope from Nikon Company.

### 1.8 RNA extraction and quantitative RT‐PCR

Total RNA was isolated from cellular samples using the Trizol reagent (Invitrogen). The reverse transcription process was facilitated by the TransScript One‐Step gDNA Removal and cDNA Synthesis SuperMix Kit (TransGen Biotech). Subsequent quantitative PCR analyses were conducted on the LC96 RT‐PCR system (Roche) utilising the TransScript Tip Green qPCR SuperMix. The qPCR protocol was initiated with a denaturation phase at 94°C for 30 s, followed by 45 amplification cycles comprising denaturation at 94°C for 5 s, annealing at 55°C for 15 s, and extension at 72°C for 10 s. Normalisation of all target gene expression was done against GAPDH levels. The relative mRNA expression was quantified using the 2^−ΔΔCT^ method. To ensure reproducibility, all procedures were executed in triplicate. Details of the primer sequences utilised are provided in Table S1.

### 1.9 Quantification of PCDHGB7 methylation by MethyLight assay

The bisulfite conversion of genomic DNA was carried out in accordance with the established protocols. We assessed the methylation status of PCDHGB7 in breast cancer tissues compared to their normal counterparts, situated at least 5 cm from the tumour, utilising a quantitative fluorescence‐based real‐time PCR assay known as MethyLight. The nucleotide sequences for the MethyLight primers and probes targeting four PCDHGB7 regions (cg00281842, cg02331883, cg00808170, cg01729977) were listed to ensure precise amplification of bisulfite‐converted DNA. Table S1 contains the primer and probe specifications.

The MethyLight assay was conducted in duplicate on an LC96 platform. Each 20 µL reaction included 2 µL of bisulfite‐treated DNA, .4 µM of both forward and reverse primers for each gene, .2 µM of the probe and 10 µL of Hieff unicon qPCR Taqman Probe Master Mix (Yeasen 1120S). The thermal cycling conditions were as follows: an initial denaturation at 95°C for 1 min, followed by 40 cycles of denaturation at 95°C for 10 s and annealing/extension at 60°C for 30 s. Methylation frequencies at specific loci were statistically determined using the 2^−△△CT^ method, with COL2A1 serving as the reference gene. All procedures were replicated thrice to ensure reliability.

### 1.10 TCGA and GTEx databases analysis

The DNA methylation prediction and PCDHGB7 mRNA expression data of breast cancers were retrieved from the Cancer Genome Atlas (TCGA) and Genotype Tissue‐Expression (GTEx) databases. We selected a total of 1085 patients, with information on basal‐like, HER2^+^, luminal A and luminal B statuses, for comparison of the PCDHGB7 mRNA levels in their respective tumours. The study also included a control group comprising 291 cases.

### 1.11 Dual‐luciferase reporter assay

For the dual‐luciferase assay, cells were seeded in 24‐well plates and subjected to transfection for 48 h. Briefly, cells were transiently co‐transfected with the MYC luciferase reporter plasmid (#11544ES03, YEASEN), the Renilla luciferase control reporter TK vector, and various plasmids tailored to specific experimental objectives. At the 48‐h mark post‐transfection, luciferase activity was measured using the Dual‐Luciferase Assay System kit (Promega), strictly adhering to the manufacturer's instructions. To ensure reliability, all procedures were performed in triplicate.

### 1.12 Transwell assay

The assessment of cell invasion and migration was conducted utilising Transwell chambers, which were equipped with polycarbonate filters featuring 8‐µm pores and coated with or without Matrigel on the upper surface. These chambers were inserted into a 24‐well plate, while the lower compartment was filled with a medium enriched with 20% FBS. Hs578T and BT549 cells, subjected to various treatments, were suspended in a serum‐free medium and seeded into the upper chamber. Following a 24‐h incubation period, cells that failed to adhere were discarded. The cells that successfully traversed and adhered to the underside of the filter were stained with crystal violet for visualisation. Quantification of the penetrated cells was performed using microscopic examination.

### 1.13 Immunohistochemistry (IHC)

The slide preparation began with deparaffinisation, followed by antigen retrieval using a specialised solution. To inhibit endogenous peroxidase activity, a 3% hydrogen peroxide solution was applied. Non‐specific staining was minimised through the application of goat serum. The slides were then incubated with the XRCC5 primary antibody (#66546‐1‐Ig, Proteintech) or PCDHGB7 primary antibody (BeiJing Yoce Biotech) at a 1:500 dilution and maintained at 4°C overnight. The following day, the sections were treated with horseradish peroxidase‐conjugated secondary antibodies specific to goat immunoglobulins and developed using DAB chromogen.

For the semi‐quantitative assessment of XRCC5 or PCDHGB7 protein expression, both the intensity and the extent of staining were considered. The intensity of staining was graded on a scale from 0 to 3, corresponding to no staining, weak (light yellow), moderate (light brown), and strong (dark brown) staining, respectively. The extent of staining was similarly graded from 0 to 4, indicating the percentage of positive staining area: less than 5%, 6%–25%, 26%–50%, 51%–75% and greater than 75%. The final expression score was derived by multiplying the intensity and extent scores, and the overall expression score was categorised as weak (+) for scores of 1–3, moderate (++) for scores of 4–6, and strong (+++) for scores of 7–12. The mean score serves as the optimal cutoff for analysis.

### 1.14 AgNOR staining

Cells cultured on coverslips were fixed using 4% paraformaldehyde for 10 min at ambient temperature. This was followed by a rinse with water, after which they were stained with a mixture composed of 50% silver nitrate solution and 2% gelatin solution in a 2:1 ratio. The cells were then incubated at a temperature of 37°C for 20 min in a dark environment. Subsequently, the slides were rinsed with distilled water and sealed with coverslips. The resulting images were examined using bright‐field microscopy, employing a Leica DM4 B microscope for image capture. Each experiment was replicated thrice, and the Image J (RRID: SCR_003070) software was utilised to quantify the percentage of AgNOR staining in each field of view.

### 1.15 Chromatin immunoprecipitation assay

A chromatin immunoprecipitation (ChIP) assay was performed following the ChIP kit manufacturer's protocol (Millipore Corporation). Briefly, 1 × 10^7 ^cells were cultured, crosslinking was performed with 1% formalin, the cells were lysed with SDS buffer, and sonication was used to fragment the DNA. An anti‐POLR1A Polyclonal antibody (Cat No.20595‐1‐AP, Proteintech) was used to precipitate the DNA–protein complex. The DNA was extracted for PCR. The sequences of rDNA Promoter, rDNA 18S and rDNA 28S PCR primers are shown in Table S1.

### 1.16 Conducting subcutaneous xenograft and lung metastatic mice models

Female BALB/c‐nude mice, aged 4–5 weeks, were acquired from Vital River Laboratory, Beijing, China. All experimental protocols involving these mice were approved by the Institutional Animal Committee of Harbin Medical University and adhered strictly to the committee's guidelines. In the subcutaneous xenograft model, each mouse in a group of eight received an injection of 1.5 × 10^6^ Hs578T cells into the flank. Tumour sizes were documented every 3 days. Mice were euthanased between 20 and 29 days post‐injection, and the tumours were subsequently excised and measured. For the lung metastasis model, 2.5 × 10^6^ Hs578T cells were administered intravenously via the tail vein to groups of 10 mice. Thirty days following the implantation, these mice were also euthanased, and their lung tissues were collected and processed for haematoxylin and eosin (HE) staining.

### 1.17 Co‐immunoprecipitation (Co‐IP) assay

The TNBC cells were transfected with either an empty vector or a PCDHGB7‐flag construct. Following a 48‐h incubation period, cells were harvested and lysed with a specialised lysis buffer to extract proteins. These protein samples were subsequently incubated with anti‐flag (specific to PCDHGB7, #20543‐1‐AP, Proteintech), or anti‐MYC antibodies (#18583S, Cell Signalling), or a control IgG (#30000‐0‐AP, Proteintech) for 24 h. To facilitate immunoprecipitation, protein A/G‐agarose beads were introduced to the mixture and allowed to incubate for an additional 24 h. The immunocomplexes captured on the beads were then resolved by SDS‐PAGE, followed by immunoblot analysis using the appropriate antibodies to detect the proteins of interest.

### 1.18 Polysome profiling

The cells underwent treatment with cycloheximide (.1 mg/mL, Sigma‐Aldrich) for 10 min at a temperature of 37°C. Subsequent to this, they were dissociated using a trypsin‐EDTA solution (.05%) for an equal timespan and subjected to a dual washing process using cycloheximide (.1 mg/mL) in 1× PBS. For cell resuspension, a polysome lysis buffer was employed, which was composed of 20 mM Tris‐HCl (pH 7.4), 5 mM MgCl_2_, 150 mM NaCl, cycloheximide (100 µg/mL), Triton X‐100 (.1%), RNase inhibitor (40 U/µL) and DNAse (24 U/mL). This was followed by a 10‐min incubation on ice and subsequent centrifugation for 10 min at 12 000 × *g*, maintaining a temperature of 4°C.

Sucrose gradients were prepared using a BioComp model 108 Gradient Master, employing 10% and 50% sucrose solutions. These solutions were prepared in RNAse‐free conditions, with sucrose dissolved in a polysomal buffer containing 15 mM Tris‐HCl (pH 7.4), 15 mM MgCl_2_ and 300 mM NaCl. The clarified supernatants from the lysed cells were then carefully layered onto the 10%–50% sucrose gradients and centrifuged at 150 000 × *g* for 180 min at 4°C, utilising a Beckman SW41Ti rotor. Post‐centrifugation, the samples were analysed with a BioComp Gradient Station, and the absorbance at 254 nm was measured using an ECONO UV detector (BioComp). This allowed for the precise calculation of the monomer to polymer ratio, which was determined by comparing the area under the curves of the monosome and polysome peaks. Ribosomal fractions were then efficiently collected using a fraction collector, concluding the process.

### 1.19 Surface sensing of translation

In this study, we employed the surface sensing of translation (SUnSET) assay, as previously reported by Goodman et al. (2011), to observe protein synthesis. Puromycin (10 µg/mL) was introduced into the cell culture medium precisely 15 and 30 min prior to cell harvesting. Subsequently, the cells were gathered and lysed using ice‐cold RIPA buffer, followed by ultrasound sonication on ice. Proteins containing puromycin were then analysed via Western blotting. The anti‐puromycin antibody utilised was procured from Millipore (Catalogue#: MABE343).

### 1.20 LC‐MS/MS analysis

For protein expression analysis, the PCDHGB7 knockdown cells compared to control cells in Hs578T cells were obtained for LC/MS. The co‐IP followed by MS was employed to identify the proteins that interacted with PCDHGB7 in TNBC cells. Briefly, the proteins were digested with trypsin, quantified, and combined with GST and MBP standard proteins in equal amounts. The tryptic peptides were reconstituted and analysed using a binary mobile phase system comprising solvent A (.1% formic acid and 2% acetonitrile in water) and solvent B (.1% formic acid in acetonitrile). A gradient programme was employed for peptide separation. Subsequently, the peptides were ionised using a capillary source and analysed by a timsTOF Pro 2 mass spectrometer. An electrospray voltage of 1.75 kV was applied. Both precursor and fragment ions were detected using the TOF detector. The timsTOF Pro operated in the data‐independent dia‐PASEF mode, capturing a full MS scan range of 300–1500 m/z and conducting 20 PASEF MS/MS scans per cycle. The MS/MS scan range was set to 400–850 m/z with an isolation window of 7 m/z.

### 1.21 Statistical analysis

The data analysis was conducted utilising SPSS software (version 20.0, RRID: SCR_002865) and Graphpad Prism (version 9.0.1, RRID: SCR_002798). We presented the findings as the mean ± standard deviation, based on three independent experiments conducted in triplicate. To compare variables, we applied Student's *t*‐test for two‐group comparisons with normally distributed data, while the Mann–Whitney *U* test was used for data not following a normal distribution. The prognostic significance of XRCC5 or PCDHGB7 levels was determined through univariate Cox regression analysis, complemented by the Kaplan–Meier method. Additionally, we used Spearman correlation analysis to explore the relationship between PCDHGB7 expression and hypermethylation regions. A *p*‐value of less than .05 was considered to indicate statistical significance.

## AUTHOR CONTRIBUTIONS

Study concept and design: **Xiaobo Li, Jing Li, Lei Zhang, Yan He** and **Guoqiang Zhang**. Acquisition and analysis: **Ming Shan, Hongjie Bi, Lili Ren, Yixin Li** and **Tianzhen Wang**. Interpretation of data for the work: **Minghui Zhang** and **Yuguang Ye**. Experiment: **Hua Sun, Zhiqi Li, Jianguo Dong, Ziyuan Wang** and **Shengbo Sang**. Writing‐original draft: **Xiaobo Li** and **Jing Li**. Writing—review and editing: **Siniša Volarević** and **Rui Su**.

## CONFLICT OF INTEREST STATEMENT

The authors declare no conflicts of interest.

## FUNDING INFORMATION

This research was supported by Natural Science Foundation of Inner Mongolia Autonomous Region of China (No. 2024MS08043); The National Natural Science Foundation of China (82273364 and 82300223); Chinese Foundation for Primary Health Care (cphcf‐2023‐018); The “Wu Jieping” Foundation (320.6750.2022‐19‐106); Science and Technology Program of the Joint Fund of Scientific Research for the Public Hospitals of Inner Mongolia Academy of Medical Sciences (2023GLLH0315); Haiyan Foundation of Harbin Medical University Cancer Hospital (JJQN2022‐09, JJZD2024‐02); The HMU Marshal Initiative Funding (HMUMIF‐21001); Beijing Innovative Medicine Development Foundation (KC2022‐JX‐0123‐03); Beijing Medical Award Foundation (YXJL‐2023‐0460‐0200).

## ETHICS STATEMENT

The application of breast cancer tissue was approved by the Institutional Ethics Committee of Harbin Medical University (KY2024‐92). All experimental protocols involving these mice were approved by the Institutional Animal Committee of Harbin Medical University and adhered strictly to the committee's guidelines.

## Supporting information



Supporting Information

Supporting Information

## Data Availability

The raw data for this study were generated at the corresponding archives; further inquiries can be directed to the corresponding authors.
