# Supplementary material for: PCDHGB7 inhibits the progression of triple‐negative breast cancer by suppressing XRCC5/MYC‐mediated ribosome biogenesis
Source: Clin Transl Med. 2025 Sep 2;15(9):e70437. doi: 10.1002/ctm2.70437 (PMC12405659; doi:10.1002/ctm2.70437)
Supplement: Supplementary file 2 — Supporting Information [file CTM2-15-e70437-s002.docx]

**Supplementary Figures**

**
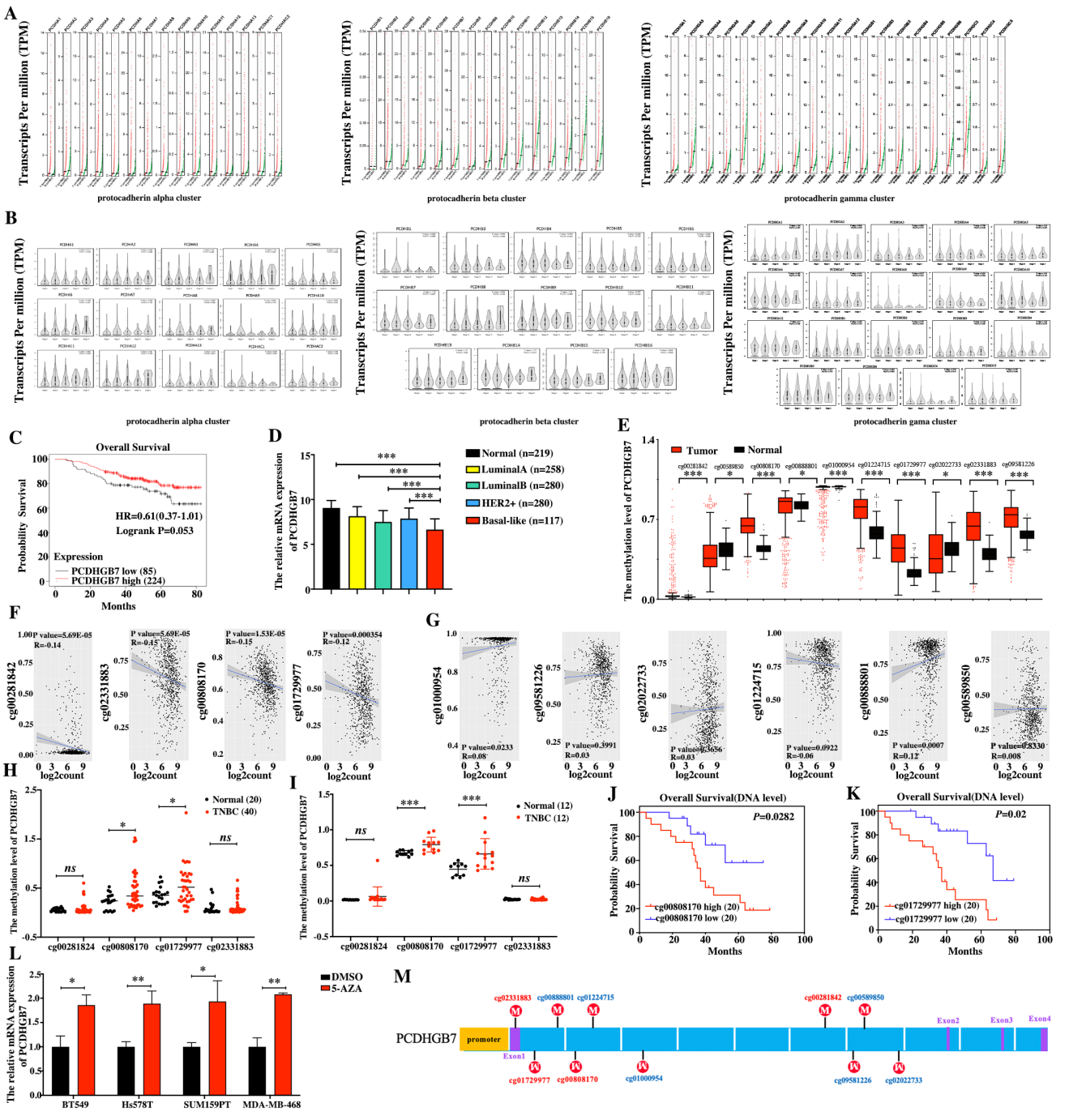
**

**Figure S1. The expression of the clustered PCDH genes in breast cancer and normal breast tissues. A.** The mRNA expression of PCDH genes in breast cancer compared with normal breast tissues was analyzed based on the GEPIA database (Tumor tissues n=1085, normal tissues n=291). **B.** The mRNA expression of PCDH genes in different stage of breast cancer was analyzed based on the GEPIA database (*p*>0.05). **C.** The correlation between the expression of PCDHGB7 and the overall survival rate of TNBC patients was analyzed using the Kaplan-Meier Plotter database (low PCDHGB7 group n=85, high PCDHGB7 group n=224, *p*=0.053). **D.** PCDHGB7 mRNA expression was analyzed and compared between normal tissues and various subtypes of breast cancer, utilizing data from both the TCGA and GTEx databases (****p*<0.001). **E.**The methylation level of different regions in PCDHGB7 was investigated in breast cancer and normal tissues based on TCGA database (Tumor tissues n=794, normal tissues n=96; **p*<0.05, ****p*<0.001). **F.** The correlation analysis between four methylation regions (cg00281842, cg02331883, cg00808170, cg01729977) in PCDHGB7 and PCDHGB7 mRNA expression based on TCGA database. **G.** The correlation analysis was examined between the methylation level of different methylation regions in PCDHGB7 promoter (cg01000954, cg09581226, cg020022733, cg01224715, cg00888801, cg00589850) and PCDHGB7 mRNA expression level based on TCGA database. **H.** The MethyLight assay was utilized to compare the methylation levels across four distinct regions between normal breast tissues and TNBC tissues (*n*=20 for normal tissues; *n*=40 for TNBC tissues; **p*<0.05, *ns* means there was no significant difference). **I.**The methylation status of four specific regions was comparatively analyzed between blood samples drawn from healthy volunteers (n=12) and those from TNBC patients (n=12) by MethyLight assay (****p*<0.001, *ns* means there was no significant difference). **J.** Kaplan-Meier survival analysis was employed to detect the impact of methylation level of cg00808170 on overall survival of TNBC patients (high cg00808170 methylation group: *n*=20, low cg00808170 methylation group: *n*=20; *p*=0.0282). **K.** Kaplan-Meier survival analysis was employed to detect the impact of methylation level of cg01729977 on overall survival of TNBC patients (high cg01729977 methylation group: *n*=20, low cg01729977 methylation group: *n*=20; *p*=0.02). **L.** PCDHGB7 mRNA expression was examined in four TNBC cell lines after 5-aza treatment (**p*<0.05, ***p*<0.01). All data are presented as means ± SD. **M.**The diagram illustrated ten different methylation regions of PCDHGB7 gene.

**
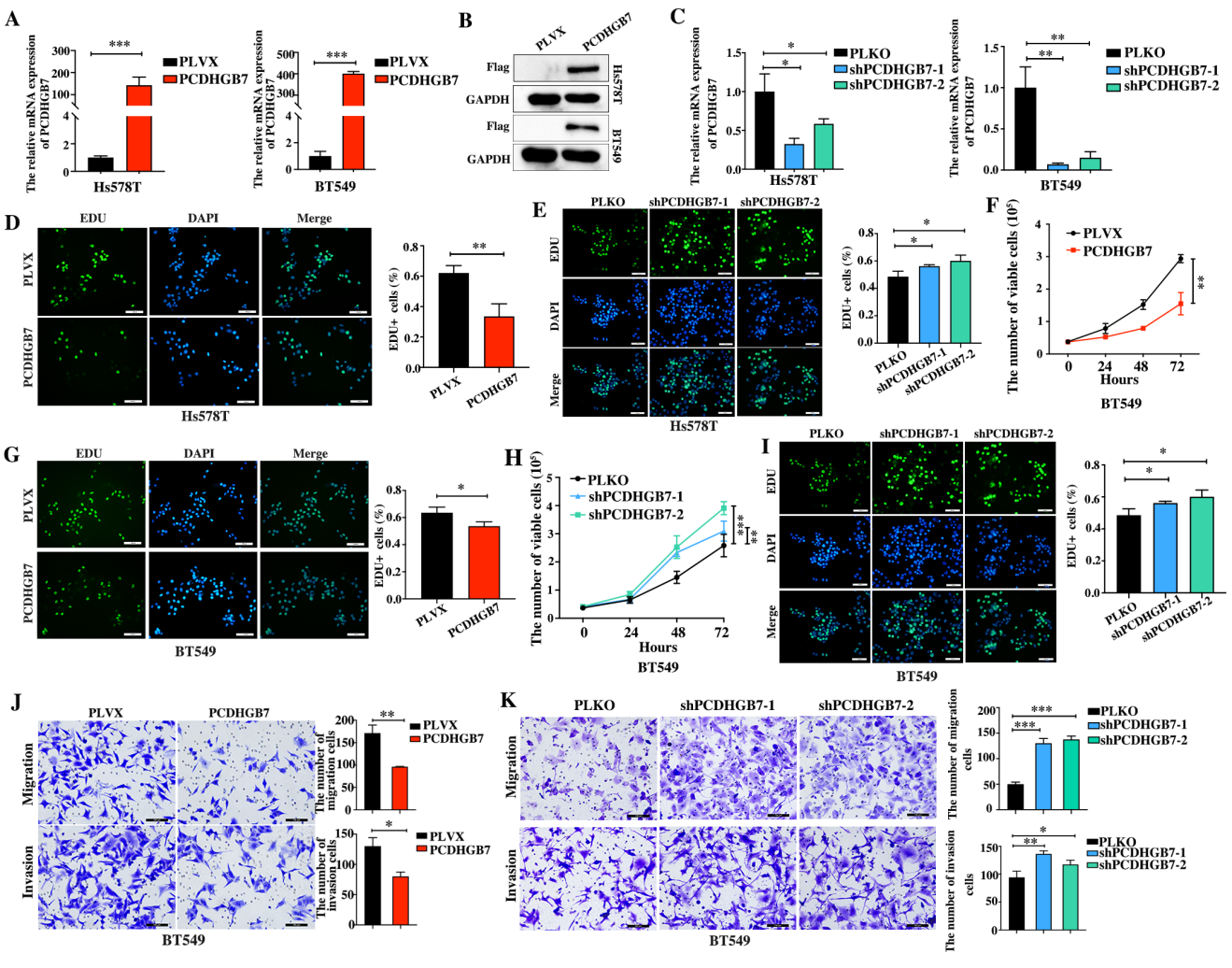
**

**Figure S2. PCDHGB7 inhibits proliferation, migration and invasion of TNBC cells. A.** The overexpression of PCDHGB7 at mRNA level in Hs578T and BT549 cells was confirmed by RT-qPCR (****p*<0.001). **B.** The overexpression of PCDHGB7 at protein level in Hs578T and BT549 cells was investigated by western blotting. **C.** The knockdown of PCDHGB7 at mRNA level in Hs578T and BT549 cells was investigated by RT-qPCR (**p*<0.05, ***p*<0.01). **D-E.**The impact of PCDHGB7 overexpression and knockdown on the proliferative capacity of Hs578T cells was assessed by EDU assay (**p*<0.05, ***p*<0.01). **F.**The impact of PCDHGB7 overexpression on cell proliferation in BT549 cells was assessed by MTT assay (***p*<0.01). **G.** The effect of PCDHGB7 overexpression on cell proliferation in BT549 cells was evaluated by EDU assay (**p*<0.05). **H.** The effect of PCDHGB7 knockdown on cell proliferation in BT549 cells was measured by MTT assay (***p*<0.01, ****p*<0.001). **I.** The effect of PCDHGB7 knockdown on cell proliferation in BT549 cells was evaluated by EDU assay (**p*<0.05). **J-K.** The effect of PCDHGB7 overexpression and knockdown on the migration and invasion of BT549 cells was examined by transwell assay (magnification, ×200; scale bars=100μm; **p*<0.05, ***p*<0.01, ****p*<0.001). All data are presented as means ± SD.

**
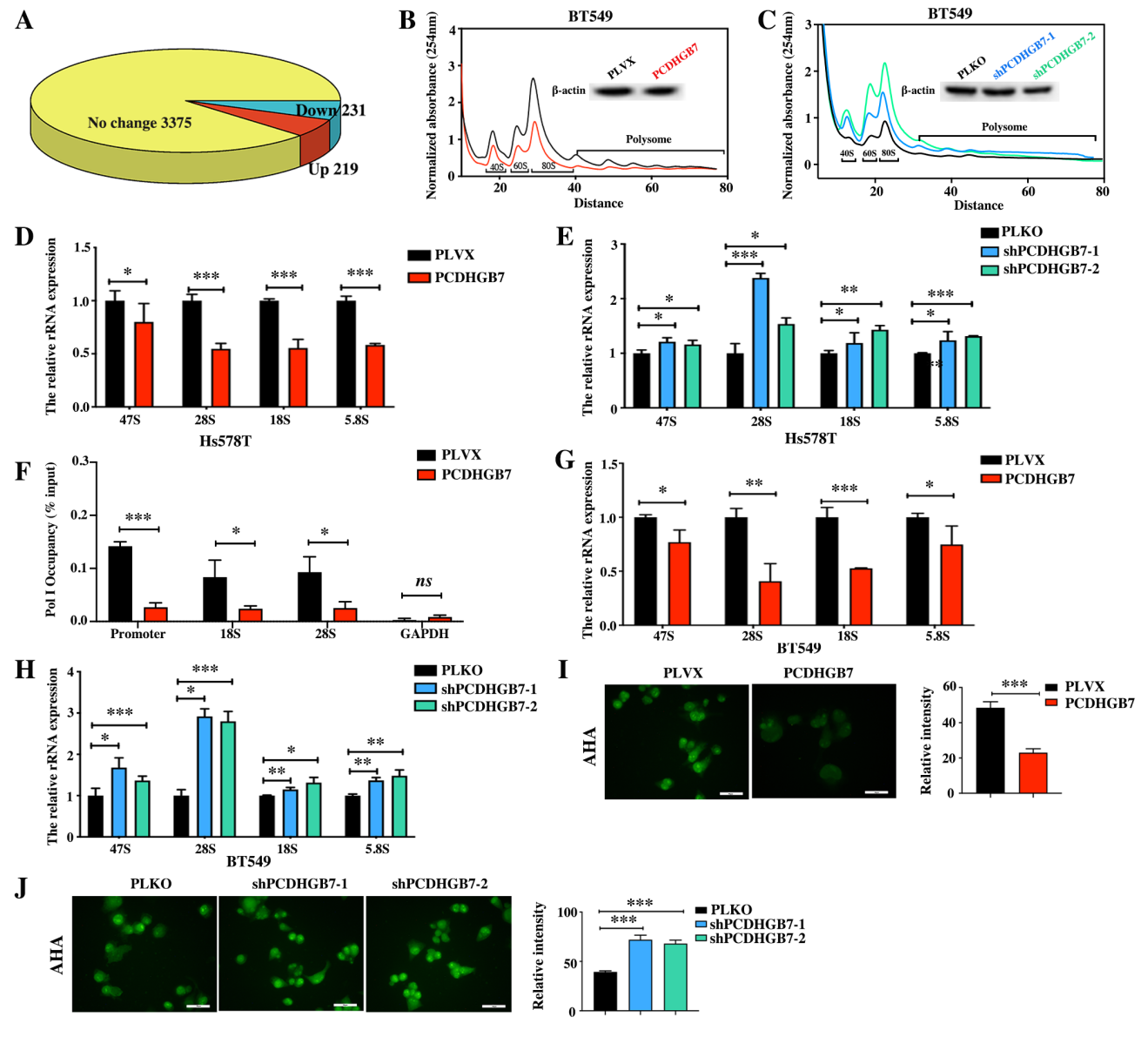
**

**Figure S3. PCDHGB7 suppresses the ribosome biogenesis in TNBC. A.** The number of significantly altered proteins upon PCDHGB7 knockdown detected by MS (fold change >1.2). **B-C.** Polysome profiling assay was performed in BT549 cells upon PCDHGB7 overexpression and knockdown. **D-E.** RT-qPCR was used to detect the level of 47S pre-rRNA and 28S, 18S and 5.8S rRNA in Hs578T cells upon PCDHGB7 overexpression and knockdown (**p*<0.05, ***p*<0.01, ****p*<0.001). **F.** ChIP assay was used to detect the change of Pol I occupancy over different regions of the rDNA in BT549-PCDHGB7 overexpression cells and control cells (**p*<0.05, ****p*<0.001, *ns* means there was no significant difference). G-H. RT-qPCR was used to detect the level of 47S pre-rRNA and 28S, 18S and 5.8S rRNA in BT549 cells upon PCDHGB7 overexpression and knockdown (**p*<0.05, ***p*<0.01, ****p*<0.001). **I-J.** AHA assay was used to detect protein synthesis in BT549 cells upon PCDHGB7 overexpression and knockdown (magnification, ×400; scale bars=50μm; ****p*<0.001). All data are presented as means ± SD.


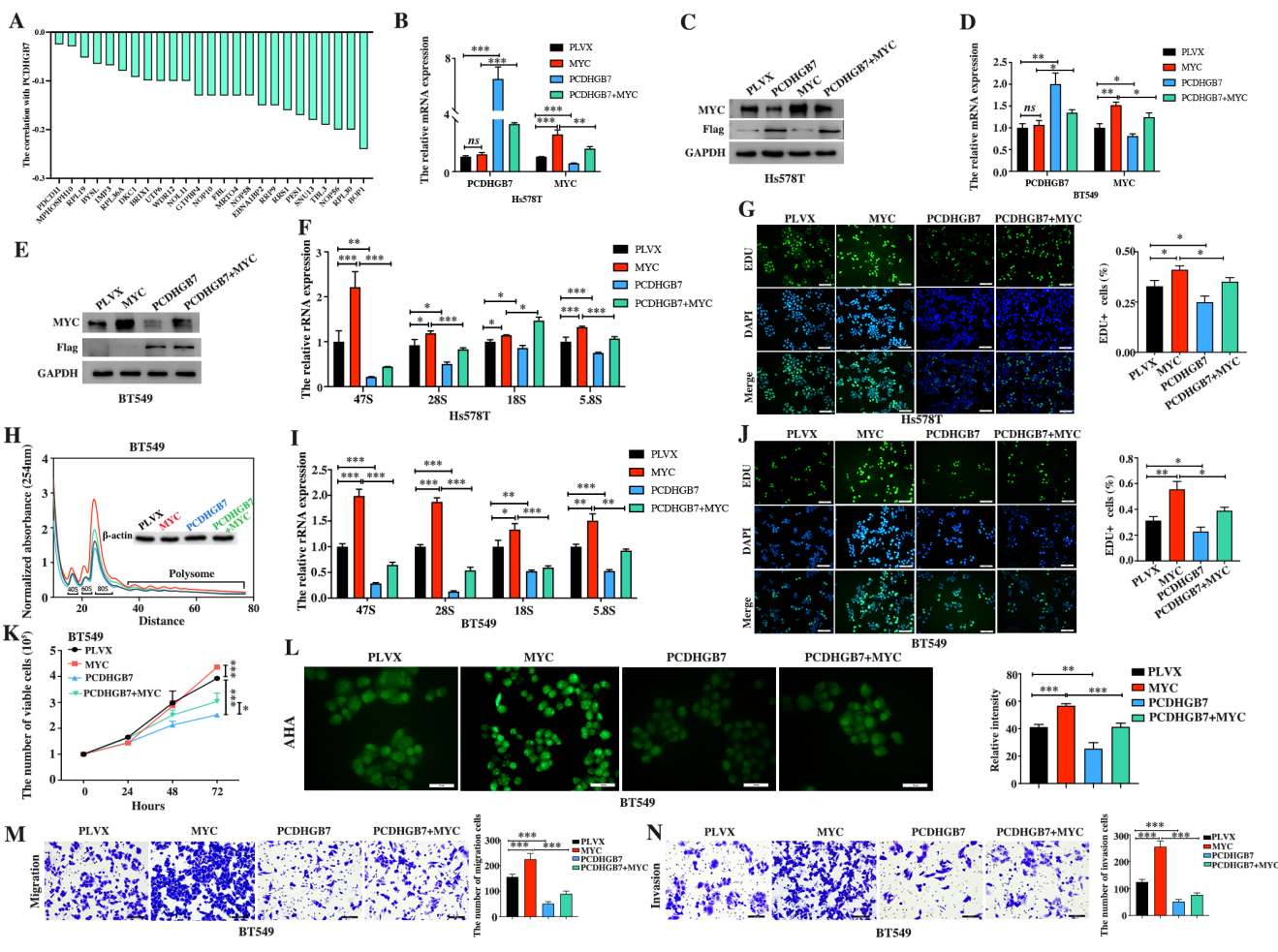


**Figure S4. PCDHGB7 suppresses MYC activity in TNBC cells. A.** The correlation analysis between the mRNA expression of PCDHGB7 and the genes enriched in the process of ribosome biogenesis and rRNA processing upon PCDHGB7 knockdown in the breast cancer tissues by GEPIA database (tumor n=1085). **B.** RT-qPCR was used to detect the expression of PCDHGB7 and MYC in Hs578T cells (****p*<0.001). **C.** Western blotting was used to detect the expression of PCDHGB7 and MYC in Hs578T cells. **D.** RT-qPCR was used to detect the expression of PCDHGB7 and MYC in BT549 cells (**p*<0.05, ***p*<0.01). **E.** Western blotting was used to detect the expression of PCDHGB7 and MYC in BT549 cells**. F.** RT-qPCR was used to detect the effect of MYC restoration on the PCDHGB7-inhibited rRNA processing in Hs578T cells (**p*<0.05, ***p*<0.01, ****p*<0.001). **G.** The effect of MYC restoration on the PCDHGB7-mediated suppression of proliferation in BT549 cells by EDU assay (magnification, ×200; scale bars=100μm; **p*<0.05). **H.** Polysome profiling assay was performed to detect the effect of MYC restoration on the PCDHGB7-mediated suppression of ribosome biogenesis in BT549 cells. **I.** RT-qPCR was used to detect the effect of MYC restoration on the PCDHGB7-mediated suppression of rRNA processing in BT549 cells (**p*<0.05, ***p*<0.01, ****p*<0.001). **J-K.** The effect of MYC restoration on the PCDHGB7-mediated suppression of proliferation in BT549 cells by EDU (**J**) and MTT (**K**) assay respectively (magnification, ×200; scale bars=100μm; **p*<0.05, ***p*<0.01, ****p*<0.001). **L.** AHA assay was used to detect the effect of MYC restoration on the PCDHGB7-mediated suppression of protein synthesis in BT549 cells (magnification, ×400; scale bars=50μm; ***p*<0.01, ****p*<0.001). **M-N.** The effects of MYC restoration on the PCDHGB7-mediated suppression of migration and invasion of BT549 cells by transwell assay (magnification, ×200; scale bars=100μm; ****p*<0.001). All data are presented as means ± SD.


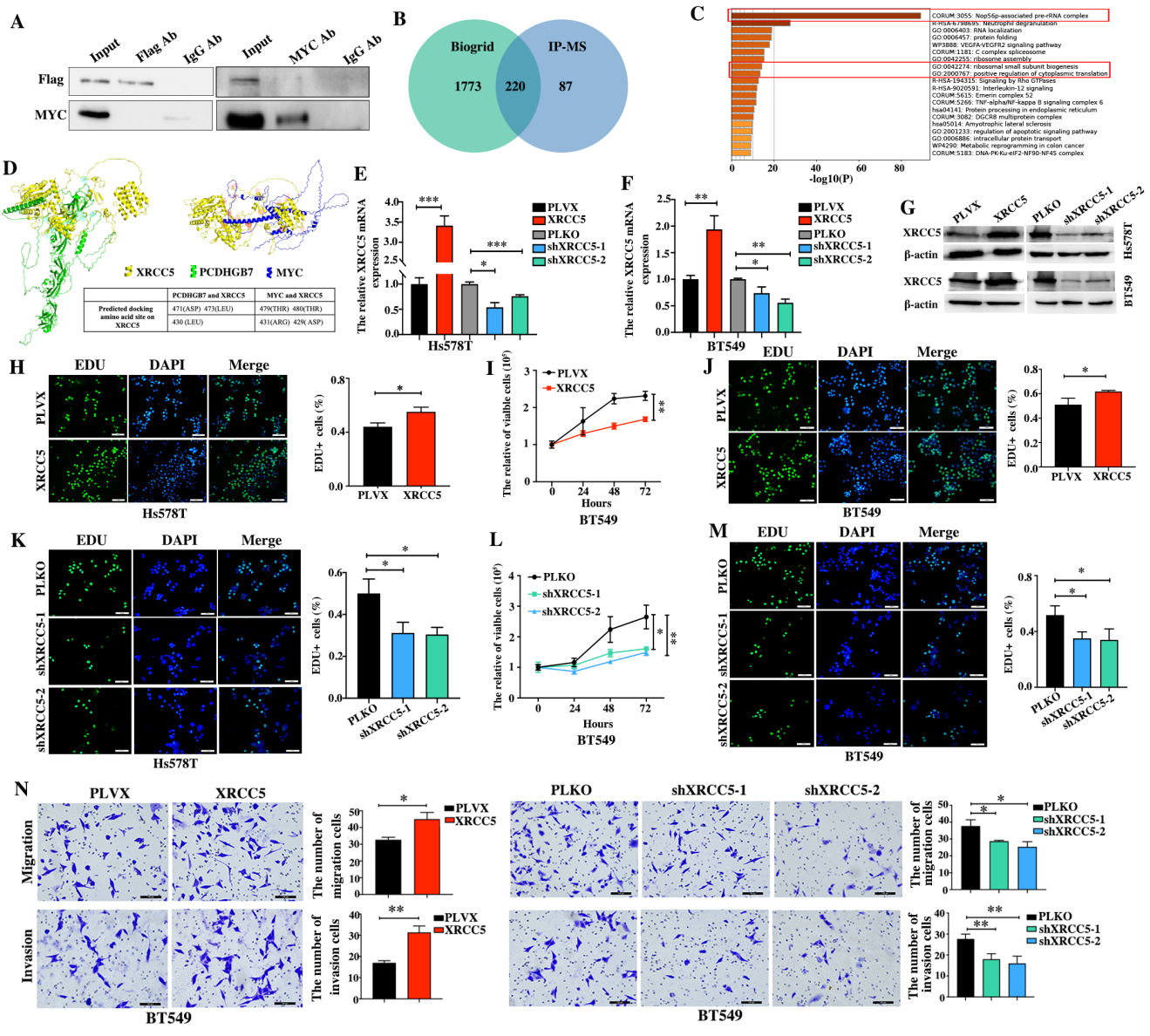


**Figure S5. PCDHGB7 suppresses MYC activity by competitively inferring XRCC5-MYC interaction in TNBC cells. A.** The interaction of PCDHGB7 and MYC was detected by co-immunoprecipitation assay. **B.** Cross-analyzing the proteins interacting with both PCDHGB7 and MYC based on MS result and Biogrid database. **C.** Functional enrichment analysis of 220 proteins interacting with PCDHGB7 and MYC was conducted using Metascape. **D.** The interaction between XRCC5 and MYC, as well as between XRCC5 and PCDHGB7, was mimicked by using GRAMM docking software. The docking amino acid sites between XRCC5 and MYC or between XRCC5 and PCDHGB7 were predicted by using GRAMM docking software. **E-F.** The overexpression or knockdown of XRCC5 in Hs578T and BT549 cells at mRNA level was investigated by RT-qPCR (**p*<0.05, ***p*<0.01, ****p*<0.001). **G.** The overexpression or knockdown of XRCC5 in Hs578T and BT549 cells at protein level was investigated by western blotting. **H.** The effect of XRCC5 overexpression on cell proliferation of Hs578T cells was evaluated by EDU assay (**p*<0.05). **I.** The effect of XRCC5 overexpression on cell proliferation of BT549 cells was measured by MTT assay (**p*<0.05). **J.** The effect of XRCC5 overexpression on cell proliferation of BT549 cells was evaluated by EDU assay (**p*<0.05). K**.** The effect of XRCC5 knockdown on cell proliferation of Hs578T cells was evaluated by EDU assay (**p*<0.05). **L.** The effect of XRCC5 knockdown on cell proliferation of BT549 cells was measured by MTT assay (**p*<0.05, ***p*<0.01). **M.** The effect of XRCC5 knockdown on cell proliferation of BT549 cells was evaluated by EDU assay (magnification, ×200; scale bars=100μm; **p*<0.05). **N.** The effect of XRCC5 overexpression and knockdown on the migration and invasion of BT549 cells was examined by transwell assay (magnification, ×200; scale bars=100μm; **p*<0.05, ***p*<0.01).


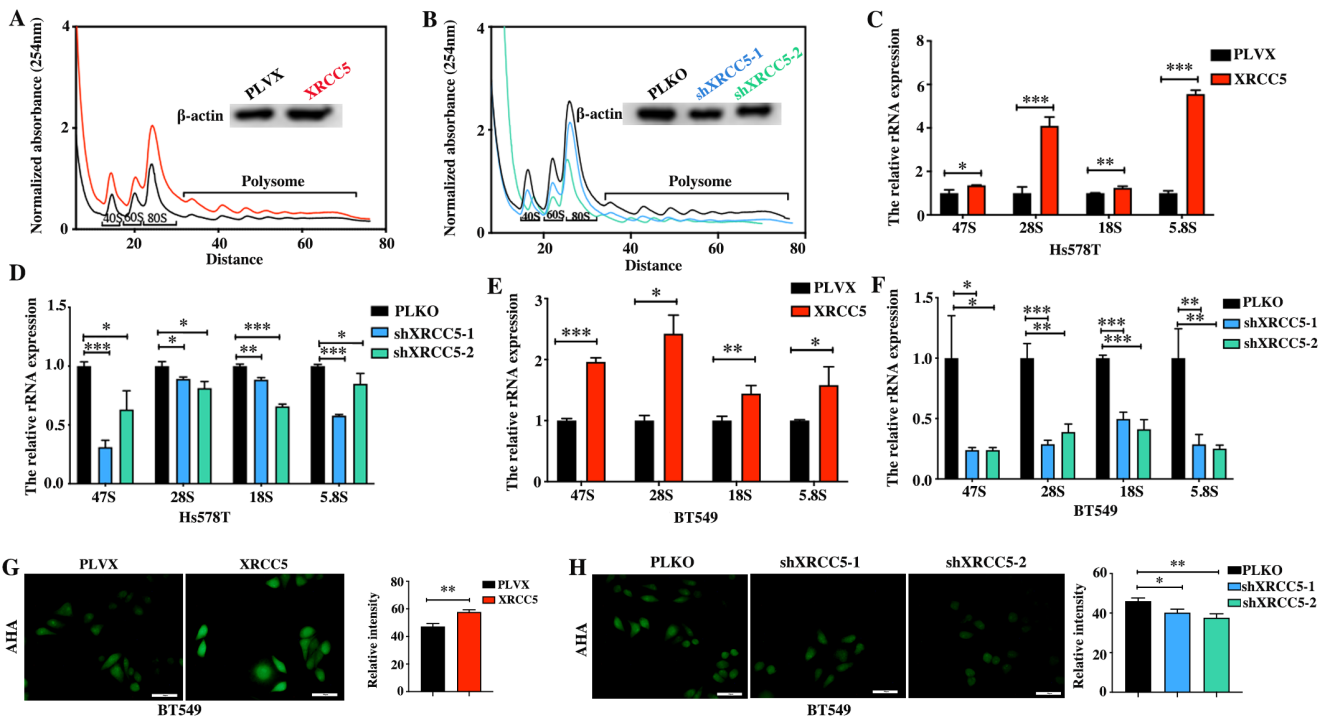


**Figure S6. XRCC5 promotes ribosome biogenesis in TNBC.**

**A-B.** Polysome profiling assay was performed in BT549 cells upon XRCC5 overexpression or knockdown. **C-D.** RT-qPCR was used to detect the 47S pre-rRNA and 28S, 18S and 5.8S rRNA in Hs578T cells upon XRCC5 overexpression or knockdown (**p*<0.05, ***p*<0.01, ****p*<0.001). **E-F.** RT-qPCR was used to detect the 47S pre-rRNA, 28S, 18S and 5.8S rRNA in BT549 cells upon XRCC5 overexpression or knockdown (**p*<0.05, ***p*<0.01, ****p*<0.001). **G-H.** AHA assay was used to show nascent peptide synthesis in BT549 cells upon XRCC5 overexpression or knockdown (magnification, ×400; scale bars=50μm; **p*<0.05, ***p*<0.01). All data were presented as means ± SD.


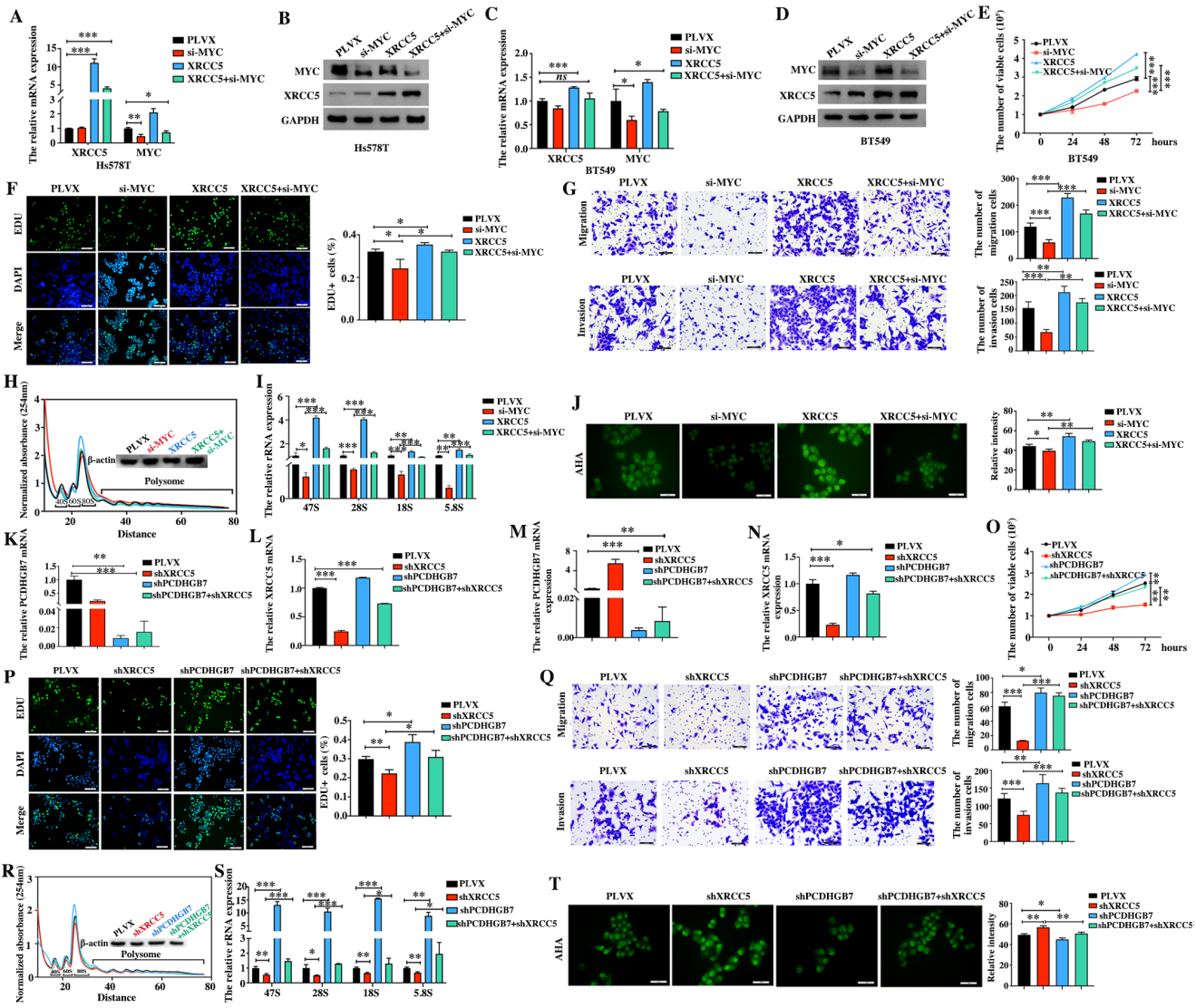


**Figure S7. PCDHGB7 inhibits the progression and ribosome biogenesis of TNBC by suppressing XRCC5-enhanced MYC activity. A-B.** The expression of MYC and XRCC5 in Hs578T cells were investigated by RT-qPCR and western blotting respectively (**p*<0.05, ***p*<0.01, ****p*<0.001). **C-D.** The expression of MYC and XRCC5 in BT549 cells was investigated by RT-qPCR and western blotting respectively (**p*<0.05, ****p*<0.001, *ns* means there was no significant difference). E. The effect of MYC knockdown on the XRCC5-induced proliferation of BT549 cells detected by MTT assay (****p*<0.001). F. The effect of MYC knockdown on the XRCC5-induced proliferation of BT549 cells was evaluated by EDU assay (magnification, ×200; scale bars=100μm; **p*<0.05). **G.** The effect of MYC knockdown on the XRCC5-induced migration and invasion of BT549 cells was assessed by transwell assay (magnification, ×200; scale bars=100μm; ***p*<0.01, ****p*<0.001). **H.** Polysome profiling assay was performed to detect the effect of MYC knockdown on the XRCC5-mediated induction of ribosome biogenesis in BT549 cells. **I.** RT-qPCR was used to detect the effect of MYC knockdown on the XRCC5-mediated induction of 47S pre-rRNA and 28S, 18S and 5.8S rRNA in BT549 cells (**p*<0.05, ***p*<0.01, ****p*<0.001). **J.** AHA assay was used to detect the effect of MYC knockdown on the XRCC5-mediated induction of nascent peptide synthesis in BT549 cells (magnification, ×400; scale bars=50μm; **p*<0.05, ***p*<0.01). **K-L.** The mRNA expression of PCDHGB7 and XRCC5 in Hs578T cells was investigated by RT-qPCR ( ***p*<0.01, ****p*<0.001). **M-N.** The mRNA expression of PCDHGB7 and XRCC5 in BT549 cells was investigated by RT-qPCR (**p*<0.05, ** *p*<0.01, ****p*<0.001). **O.** The impact of XRCC5 knockdown on the PCDHGB7 knockdown-induced proliferation of BT549 cells, as detected by MTT assay (***p*<0.01). **P.** The effect of XRCC5 knockdown on the PCDHGB7 knockdown-induced proliferation of BT549 cells, as detected by EDU assay (magnification, ×200; scale bars=100μm; **p*<0.05, ***p*<0.01). **Q.** The impact of XRCC5 knockdown on the PCDHGB7 knockdown-induced migration and invasion of BT549 cells, as detected by transwell assay (magnification, ×200; scale bars=100μm; **p*<0.05, ***p*<0.01, ****p*<0.001). **R.** Polysome profiling assay was conducted to assess the impact of XRCC5 knockdown on PCDHGB7 knockdown-induced ribosome biogenesis in BT549 cells. **S.** RT-qPCR was used to detect the effect of XRCC5 knockdown on the PCDHGB7 knockdown-induced production of 47S pre-rRNA and 28S, 18S and 5.8S rRNA in BT549 cells (**p*<0.05, ***p*<0.01, ****p*<0.001). **T.** AHA assay was used to show the effect of XRCC5 knockdown on the PCDHGB7 knockdown-induced protein synthesis in BT549 cells (magnification, ×400; scale bars=50μm; **p*<0.05, ***p*<0.01). All data were presented as means ± SD.
